# Supplementary material for: Prevalence, patterns and correlates of smokeless tobacco use in Nigerian adults: An analysis of the Global Adult Tobacco Survey
Source: PLoS One. 2021 Jan 6;16(1):e0245114. doi: 10.1371/journal.pone.0245114 (PMC7787524; doi:10.1371/journal.pone.0245114)
Supplement: Attachment — Submitted filename: Response to Reviewers.docx [file pone.0245114.s002.docx]

Nigeria Field Epidemiology and Laboratory Training Programme

22^nd^ December, 2020

Dear Dr Glantz,

**Response to Reviewers:** **PONE-D-20-25941**

Thank you for the careful review of our manuscript. After careful consideration of the reviews, I hereby write this rebuttal letter to respond to the points and comments raised by the reviewers.

The following are the responses to the points you raised:

A. **Response to Academic Editor:**
1. Comment:

*In your abstract you say, “However, the perception of possible harm from its use was low. We recommended targeted interventions to increase awareness of the harmful effects.” Your response to Reviewer 1 had a more specific and informative statement: “The use of the term “low levels of awareness of SLT harm” is relative to levels for manufactured cigarettes and other countries. However, as much as 68.9% of Nigerian adults, a clear majority, are aware of SLT harm. Note that awareness of harm is not the only factor driving levels of use. There are other critical drivers such as cultural and social norms, and acceptability of the habit. The key thing about awareness levels and use status from our study is that there is a clear association between lack of awareness of harm and likelihood of use.”*

*Please revise the abstract, discussion and conclusion of the paper to reflect this more sophisticated explanation. Please also discuss the “other factors” driving use and how consideration of those factors could inform prevention efforts.*

**Response:**

Adjustments have been made to reflect this more sophisticated explanation as advised. These adjustments are in the concluding part of the abstract, the paragraph spanning lines 262 to 273 of the revised unmarked manuscript and in the conclusion.

Yours sincerely,

Ikenna Onoh	MBBS, MSc

Resident, Nigeria Field Epidemiology and Laboratory Training Programme

ikeonoh@yahoo.com; +2347036354733
